# The epidemiology of childhood tuberculosis in the Netherlands: still room for prevention

**DOI:** 10.1186/1471-2334-14-295

**Published:** 2014-05-31

**Authors:** Connie GM Erkens, Gerard de Vries, Sytze T Keizer, Erika Slump, Susan van den Hof

**Affiliations:** 1 KNCV Tuberculosis Foundation, The Hague, The Netherlands; 2 Public Health Service of Amsterdam (GGD), Amsterdam, The Netherlands; 3 Department of Respiratory Epidemiology, National Institute for Public Health and the Environment, Bilthoven, The Netherlands; 4 Amsterdam Medical Center, Center for Poverty-Related Communicable Diseases, Amsterdam, The Netherlands

**Keywords:** Tuberculosis, Latent tuberculosis infection, Children, Preventive therapy, Contact investigation, Prevention

## Abstract

**Background:**

The occurrence of tuberculosis (TB) among children has long been neglected as a public health concern. However, any child with TB is a sentinel event indicating recent transmission. Vaccination, early case finding and treatment of those latently infected with TB can prevent cases, severe morbidity and unnecessary death.

**Method:**

The objective of the study was to describe the occurrence of TB events among children in the Netherlands which may be avoided through preventive measures. For this purpose we performed a trend analysis of routine Dutch TB and LTBI (surveillance data in 1993–2012 and a descriptive analysis of children with TB and with LTBI diagnosed in 2005–2012).

**Results:**

Overall childhood TB incidence has declined over the last two decades from 3.6 in 1993 to 1.9 per 100,000 children in 2012. The decline was stronger among Dutch-born children compared to foreign-born children. In 2005–2012 64% of childhood TB cases were detected through active case finding. Foreign-born children with TB were less likely to be detected through active case finding, when not detected through post-entry TB screening. Childhood TB diagnosis was culture confirmed in 68% of passively detected cases and 12% of actively detected cases. Of 1,049 children with LTBI started on preventive treatment in 2005–2012, 90% completed treatment. In 37% of all childhood TB cases there was at least one ‘missed opportunity’ for prevention. Thirty nine percent of child TB patients eligible for BCG were not vaccinated.

**Conclusion:**

Children with TB in the Netherlands are generally detected at an early stage and treatment completion rates are high. However, more TB cases among children can be prevented through enhancing TB case finding and screening and preventive treatment of latent TB infection among migrant children, and improving the coverage of BCG vaccination among eligible risk groups.

## Background

According to estimates of the World Health Organization 530,000 tuberculosis (TB) cases occurred among children in 2012, 6% of the total number of TB cases (WHO 2013). In countries in the European Union and European Economic Area, the percentage of children younger than 15 years of age among TB patients was 4.4% in 2011 (range 0–11.1%) [1]. Children with TB disease are generally not infectious, however TB infection and disease in a child can be regarded as a sentinel event for ongoing transmission. The clinical presentation of TB differs in children from adults. Symptoms and signs are often non-specific and vary from minor to life threatening. In children TB typically presents by the formation of a primary complex in the lungs consisting of a small peripheral pulmonary focus and hilar or paratracheal lymph node involvement. Bacteriological confirmation is difficult to obtain [2-6]. International standards recommend sputum induction, bronchoscopy or nasopharyngeal and gastric lavage in children to obtain two to three samples for culture and drug sensitivity testing [7-10], but children often have paucibacillary disease and samples often do not contain detectable bacteria. Childhood TB control involves diagnosis and treatment of TB disease with methods appropriate for children, systematic active case-finding among children in contact with infectious TB, therapy for children with latent TB infection (LTBI) [11] to prevent progression to disease, and Bacillus Calmette-Guérin (BCG) vaccination. BCG offers best protection if offered at a young age [12].

In the Netherlands prevention and control of TB is the responsibility of the Municipal Public Health Services (MPHS). TB public health physicians in the MPHSs diagnose and treat two thirds of children with TB [13], while one third of the children with TB is diagnosed and treated by a large number of pediatricians and pulmonologists. TB nurses of the MPHSs take care of daily observed treatment (DOT) in children with TB or TB infection or inform and instruct the parent or guardian how to administer the drugs. Prevention of TB in children focuses on 1) active case finding through screening of new immigrants, and contact investigations around an index TB patient, and 2) BCG vaccination. Additionally MPHSs offer (primary) preventive treatment to child contacts with TB infection identified in contact investigation. Until 2002, BCG-vaccinated TB contacts, including children, were screened only with chest X-ray and were not eligible for LTBI screening [14]. In 2002, LTBI screening using TST was introduced for all children, including those BCG-vaccinated. From 2007, LTBI screening using interferon gamma release assays (IGRA) in TST-positive contacts (two step approach) was recommended [15].

In the Netherlands BCG vaccination is targeted to new-born children with a parent born in a country with estimated TB incidence >50 per 100,000 population. Immigrant children younger than 12 years of age, with no evidence of BCG-vaccination at entry screening for TB and a negative tuberculin skin test (TST) are offered BCG-vaccination. Non-BCG-vaccinated children with a positive TST are offered preventive treatment for latent tuberculosis infection (LTBI).

The effectiveness of TB control efforts among children in the Netherlands has not been evaluated before. This study aims to describe trends and characteristics of TB and LTBI among children and to evaluate TB control efforts through the identification of risk factors for delayed case finding and exploring opportunities where prevention can be further improved.

## Methods

Data from all notified TB and LTBI cases in 1993–2012 were retrieved from the Netherlands TB Register (NTR). All clinical and bacteriologically confirmed TB cases are mandatory notified to the MPHSs. Since 1993 the MPHSs report all mandatory notified TB cases in a central registry. Additionally, they voluntarily report all new LTBI cases diagnosed and eligible for preventive treatment. The web-based notification process for both TB and LTBI cases, has an average timeline of 6–12 months and consists of four parts: 1) notification, 2) demographic and clinical characteristics, including laboratory confirmation and DNA fingerprint results, 3) patient management and (preventive) treatment outcome and 4) results of contact investigation (from 2006 onwards). Registered data include method of case finding and ethnic origin.

We analyzed the trend in incidence for all notified TB and LTBI cases in the period 1993–2012 comparing TB incidence among children younger than 15 years of age with TB incidence among adults, stratified by ethnic group defined as foreign-born, native Dutch (born in the Netherlands and both parents born in the Netherlands) and second generation immigrant (born in the Netherlands and at least one parent foreign-born). Population data for the calculation of incidence rates were obtained from the Office of National Statistics, Statistics Netherlands (CBS). CBS population data on ethnic groups are available from 1996 onwards. Linear trend analysis was done in Excel (Microsoft Office 2007).

We described characteristics by age group and method of case finding for childhood TB cases notified in the period 2005–2012. This period was selected to describe the more recent cases and avoid registration bias, as the content of the data collection questionnaire was changed in 2005. We conducted logistic regression analysis in SPSS 19.0 (Chicago, IL, USA) to identify person and disease characteristics associated with passive case finding as a proxy for delayed case finding. Variables yielding a p-value <0.2 in univariate analysis were included in multivariate analysis, and the most parsimonious model was selected by backward elimination guided by the change in log likelihood and coefficients of successive models.

Opportunities in prevention were categorized according to three levels of opportunities for prevention: primary) vaccination and source case investigation, secondary) identification and preventive treatment of children with LTBI and tertiary) early case finding and treatment completion. As outcome indicators we chose coverage of the existing preventive interventions (BCG vaccination and contact investigation) among children identified with TB and LTBI, percentage of children with TB identified through entry screening among the target group, and unsuccessful completion rates of both preventive and curative treatment. Children with TB who potentially could have been identified with LTBI through screening on entry and offered preventive treatment, were also included as an indicator of opportunity for prevention, although at the time of the registration of the cases, the national guideline only recommended this screening in non-BCG vaccinated children.

## Results

### Trend analysis childhood TB incidence 1993–2012

**Figure 1:**
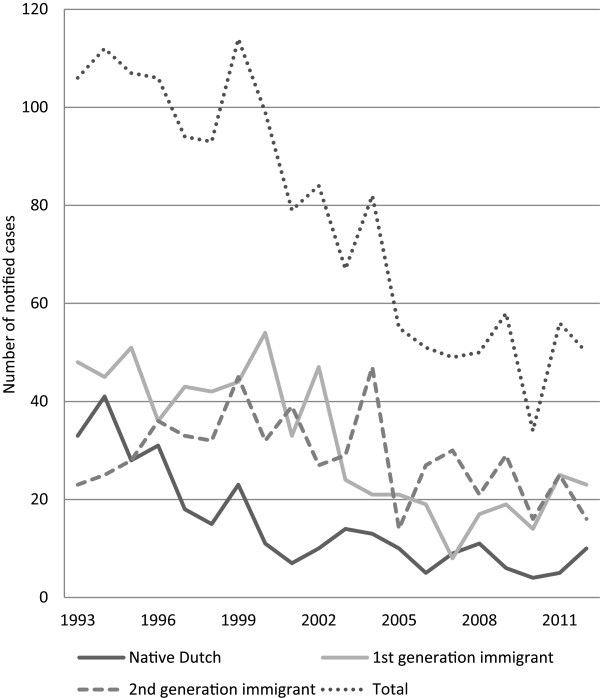
Tuberculosis in children <15 years by ethnic origin in the Netherlands 1993–2012.

**Figure 2:**
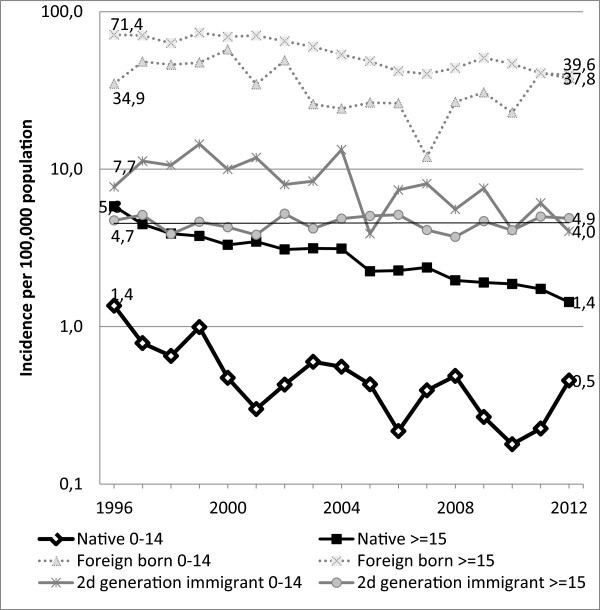
Trend in tuberculosis incidence in children by ethnic origin in the Netherlands, 1996–2012.

The absolute number of children with TB decreased from 106 in 1993 to 50 in 2012, with a corresponding incidence of 3.6 and 1.9 per 100,000 population (p < 0.001). From 1993 until 2000 there was no clear change in the annual incidence. Since 2001 the average annual decline in childhood TB cases was 2.6%. The percentage of child TB cases among all cases declined from 6.7% in 1993 to 5.2% in 2012 (p = 0.001). The incidence rate declined markedly from 1996–2012 among native Dutch children (1.4 to 0.5 per 100,000 (p < 0.001)), native Dutch adults (5.8 to 1.4 per 100,000 (p < 0.001) and foreign-born adults (71.4 to 39.6 per 100,000 (p = 0.003) (Figures 1 and 2). No significant trends were observed among foreign-born children (34.9 to 37.8 per 100,000, p = 0.81), among second generation children (7.7 to 6.1 per 100,000, p = 0.38), and among second generation adults (4.7 to 4.0 per 100,000, p = 0.53).

### Characteristics of children with TB 2005–2012

In summary, 403 children with TB disease were registered, 5% of all notified cases in 2005–2012 (Table 1). Of all children with TB, 56 (14%) were younger than 2 years and 98 (24%) were 2–4 years old. Child TB patients were more often born in the Netherlands than adult patients, but 60% of the children were second generation immigrants. Foreign-born children with TB were mostly born in Somalia (46%) and other sub-Sahara African countries (24%). The most common forms of TB among children were primary TB infection (35%) and pulmonary TB (38%). Two children aged 10–14 years were diagnosed with multi-drug resistant TB. No relapse TB cases among children were observed.

**Table 1:** Characteristics of TB cases in the Netherlands 2005-2012

	**< 5 years**		**5-14 years**		**Total 0–14 years**		**>14 years**		**Total (all ages)**	
	**n**	**% of age group**	**n**	**% of age group**	**n**	**% of age group**	**n**	**%**	**n**	**% of age group**
Total TB cases *(% of total notified)*	154	*(2%)*	249	*(3%)*	*403*	*(5%)*	7954	*(95%)*	8357	*(100%)*
Male	82	53%	112	45%	194	48%	4574	58%	4768	57%
Dutch born, including both parents	33	21%	69	28%	102	25%	1914	24%	2016	24%
Dutch born, 2d generation immigrant	91	59%	63	25%	154	38%	381	5%	535	6%
Foreign-born	30	19%	116	47%	146	36%	5,645	71%	5791	69%
*WHO incidence 2008 country of origin*										
<*50*	*4*	*13%*	*8*	*7%*	*12*	*8%*	*870*	*15%*	*882*	*16%*
*50-199*	*7*	*23%*	*24*	*21%*	*31*	*21%*	*2673*	*47%*	*2704*	*48%*
>*200*	*19*	*63%*	*84*	*70%*		*68%*	*2116*	*37%*	*2185*	*39%*
Site of TB disease										
- Primary TB infection	72	47%	69	28%	141	35%	117	1%	258	3%
- Pulmonary TB	51	33%	86	35%	137	34%	4189	53%	4326	52%
- TB pleuritis	1	1%	7	3%	8	2%	560	7%	568	7%
- Other TB of respiratory tract, including intrathoracic lymph nodes	15	10%	35	14%	50	12%	896	11%	946	11%
- TB of peripheral lymph nodes	7	5%	32	13%	39	10%	1260	16%	1299	16%
- miliary TB	3	2%	7	3%	10	2%	203	3%	213	3%
- TB meningitis	3	2%	4	2%	7	2%	177	2%	184	2%
- Bone and joint TB	0	0%	5	2%	5	1%	341	4%	346	4%
- TB of other organ tracts	2	1%	10	4%	12	3%	732	9%	744	9%
Culture positive	36	23%	93	37%	129	32%	5,867	74%	4611	55%
History of TB contact	105	68%	132	53%	237	59%	606	8%	843	10%
Hiv-co-infection	1	1%	2	1%		1%	326	4%	329	4%
Diagnosis by MPHS	104	68%	140	56%	244	61%	1509	19%	1753	21%
Active case finding										
- Contact investigation	101	66%	128	51%	229	57%	390	5%	619	7%
- Screening immigrants	11	7%	18	7%	29	7%	856	11%	885	11%
Hospital admission >1 week	23	15%	41	16%	64	16%	2652	33%	2716	32%
Daily observed treatment	88	57%	116	47%	204	51%	2114	27%	2318	28%
Adverse events*	4	3%	12	5%	16	4%	1022	13%	1038	12%
Treatment Outcome	135		218		353		7046		7399	
- Success	124	92%	207	95%	331	94%	6070	86%	6401	87%
- Defaulted	5	4%	3	1%	8	2%	255	4%	263	4%
- Death caused by TB	2	1%	1	0%	3	1%	135	2%	138	2%
- Death caused by other cause	1	1%	0	0%	1	0.3%	271	4%	272	4%
- Transfer out/unknown	3	2%	7	3%	10	3%	315	4%	325	4%

*Definition of abbreviations*: *NL* Netherlands, *CI* contact investigation, *MPHS* Municipal Public Health Service, *HIV* human immunodeficiency virus, *TB* tuberculosis, *BCG* Bacillus Calmette-Guérin.

*Adverse events- defined as events demanding an interruption or change of the treatment regimen.

Overall, 32% of childhood TB diagnoses were confirmed with a positive culture; 68% of passively detected cases and 12% of actively detected cases. In passively detected patients, culture was performed in 117/145 (81%) cases and 98/117 (84%) were culture positive. In contrast, culture was performed in 74/258 (29%) actively detected cases and of these cases 31/74 (41%) were culture positive. After adjustment for ethnicity and type of TB, the association between passive case finding and culture performance was still present (OR adj. 2.6, 95% CI 1.2-5.3).

### Treatment outcome

Fifteen percent of children with TB were admitted to a hospital compared with 33% of adult TB cases (Table 1). Treatment was more often administered under DOT in children (51%) than in adults (28%). In total 94% of the children completed TB treatment with success, compared with 86% of adults. Few child TB cases (2%) interrupted the treatment: five children younger than 5 years of age and three children older than 5 years. Three children died due to TB and one from other causes. Serious forms of TB were associated with a high risk of mortality: two of the seven children with miliary TB died from TB.

### Trends in diagnosis of LTBI

**Figure 3:**
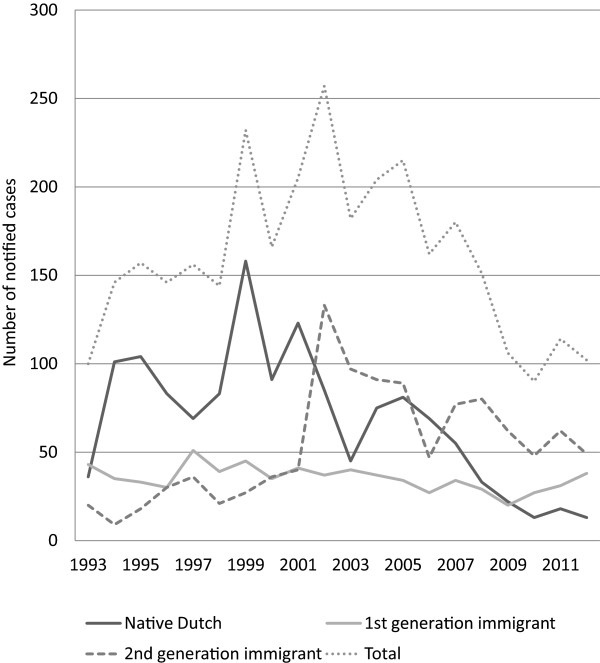
Latent tuberculosis infection in children < 15 years by ethnic origin in the Netherlands 1993–2012.

From 1993 to 2002 the annual number of children diagnosed and reported with LTBI increased from 100 to 257, an average increase of 17% per year. After 2002, the number of LTBI reports declined to 102 in 2012. This decrease was most marked among native Dutch children and second generation immigrant children: the number of LTBI diagnoses decreased with 85% and 63% respectively (Figure 3). Among foreign-born children, the number of LTBI diagnoses remained more or less stable.

### Characteristics of children with LTBI

In the period 2005–2012 1120 children with LTBI were notified, 8% of all notified persons with LTBI (Table 2). Overall 21% of these children were foreign-born and 46% were second generation migrants. The majority (83%) of children with LTBI was diagnosed in the context of contact investigations. This proportion was lower among foreign-born children (55%) who relatively more often were detected through entry screening (18%) and before BCG vaccination (27%) (data not shown). The acceptance rate of preventive treatment was higher in children (94%) compared to adults (72%) (p < 0.001). Children starting preventive treatment also completed the treatment significantly more often than adults (90% versus 81%, p < 0.001).

**Table 2:** Characteristics of persons notified with LTBI 2005–2012

	**< 5 years**		**5-14 years**		**Total 0–14 years**		**>14 years**		**Total (all ages)**	
	**n**	**% of age group**	**n**	**% of age group**	**n**	**% of age group**	**n**	**% of age group**	**n**	**% of age group**
Total new LTBI cases *(% of total notified)*	298	*(2%)*	822	*(6%)*	1120	*(8%)*	12121	*(92%)*	13241	*(100%)*
Ethnic origin										
- Native Dutch	43	14%	261	32%	304	27%	6820	56%	7124	54%
- Foreign-born	50	17%	190	23%	240	21%	2,899	24%	3139	24%
- 2d generation immigrant	186	62%	328	40%	514	46%	607	5%	1121	8%
- Unknown*	19	6%	43	5%	62	6%	1795	15%	1857	14%
Detected through contact investigation	227	76%	708	86%	935	83%	6957	57%	7892	60%
Preventive treatment started	283	95%	766	93%	1049	94%	8669	72%	9718	73%
- Of whom completed	249	*(88%)*	690	*(90%)*	939	*(90%)*	7009	*(81%)*	7948	*(82%)*
*(% of preventive treatment)*										
-Defaulter	25	*(9%)*	47	*(6%)*	72	*(7%)*	1157	*(13%)*	1229	*(13%)*
-Transfer out/unknown	9	*(3%)*	29	*(4%)*	38	*(4%)*	497	*(6%)*	535	*(6%)*

*Country of birth is unknown in 106 persons among whom 3 children country and of origin of the parents is unknown in 1505 persons among whom 53 children. *Definition of abbreviations*: *NL* Netherlands, *BCG* Bacillus Calmette-Guérin.

### Opportunities for prevention

An overview of the opportunities of TB control is given in Table 3. In 37% of all TB cases there was a ‘missed’ opportunity for prevention.

**Table 3:** Indicators of (missed) opportunities in childhood tuberculosis control 2005-2012

**Opportunity**	**Indicator**	**Number**	**Total target group for the intervention**	**% of target group**
***Primary prevention: *** **BCG vaccination and infection prevention**	Children with LTBI belonging to target groups of BCG vaccination without BCG vaccination	163	474	34%
	Children with TB belonging to target groups of BCG vaccination without BCG vaccination	83	212	39%
	Children with TB meningitis or miliary TB belonging to target groups of BCG vaccination without evidence of BCG vaccination	7	9	78%
	Children with passively detected TB and no known source case and no source or contact investigation	26	153	17%
***Secondary prevention: *** **screening and preventive treatment of LTBI**	Foreign-born children with TB 7–30 months after entry in the Netherlands and detected passively through the development of symptoms	27	45	60%
	Children with TB reported with a history of contact with infectious TB patient but not detected through contact investigation	16	237	7%
	Children with LTBI not started on preventive treatment	71	1120	6%
	Children not completing preventive treatment	85	1024	8%
***Tertiary prevention: *** **early case finding and adequate treatment of TB**	Foreign-born children with TB < 6 months after entry in the Netherlands and detected passively through the development of symptoms	15	48	31%
	Children with TB with an unfavorable outcome of TB treatment	14	375	4%
Overall*	Children with TB and at least 1 missed opportunity for prevention	150	403	37%

*Children may have more than one ‘missed’ opportunity for TB prevention. *Definition of abbreviations*: *NL* Netherlands, *TB* tuberculosis, *LTBI* latent tuberculosis infection, *BCG* Bacillus Calmette-Guérin.

In terms of opportunities for primary prevention through BCG vaccination, 212 (53%) children with TB were eligible for BCG vaccination, of whom 83 (39%) were reported as not BCG-vaccinated. Fifty-one (61%) of these children were younger than 5 years of age, of whom 46 were born in the Netherlands. Foreign-born child TB patients were more often BCG-vaccinated (54%) compared with second-generation migrant child TB patients (35%). Seventeen out of 139 (12%) children younger than 5 years and in 15/335 (4%) children aged 5–11 years at entry in the Netherlands were not BCG vaccinated. Four children with TB meningitis and five children with miliary TB belonged to the target group for BCG-vaccination, seven of these children were not vaccinated. Of 474 children eligible for BCG vaccination and diagnosed with LTBI, 163 (34%) were reported as not vaccinated with BCG.

Data on source and contact investigation (SCI) were available for 347 (86%) children. In 184 (53%) cases a SCI was not performed or unknown SCI was not undertaken in 26 (17%) passively detected cases with no known history of TB contact. When no SCI was performed the majority of the children (149 (81%)) had been detected through SCI or screening themselves.

In terms of opportunities for secondary prevention, 27 children with TB within the first 2.5 years after migration to the Netherlands, could probably have been identified with TB infection at entry screening. Furthermore, 16 child TB contacts were not identified with TB or TB infection by contact investigation. Of children identified with LTBI, 71 (6%) did not start preventive treatment and 85 (8%) did not complete treatment.

In terms of opportunities for tertiary prevention, the majority of children with TB were found through active case finding (73% in children <5 years and 59% in children 5–14 years), mostly through contact investigation. In total 15 TB cases (31% of the target group) were missed by screening at entry in the Netherlands (Table 3). Overall 3% of the children with a known treatment outcome did not complete the TB treatment successfully.

The proportion of cases detected through active case finding was lowest among foreign-born children with length of stay 7–30 months (40%) and >2.5 years (41%) in the Netherlands (Table 4). These selected groups showed a significantly higher risk to be detected through passive case finding compared to native Dutch children, after adjustment for type of TB (OR adj. 3.9, 95% CI 1.4-10.7 and 2.7, 95% CI 1.0-6.8 respectively).

**Table 4:** Characteristics of childhood TB cases in the Netherlands 2005–2012 according to method of case finding, and characteristics associated with risk to be detected with passive case finding

				**Method of case detection**		**Unadjusted OR (95% CI)**	**Adjusted**^**# **^**OR (95% CI)**
				**Active case finding**	**Passive case finding**		
				**Number (row%)**	**Number (row%)**		
**Total**				**258 (64%)**	**145 (36%)**		
Age group	0-1 years			39 (70%)	17 (30%)	0.40 (0.21-0.77)	
	2-4 years			73 (74%)	25 (26%)	0.31 (0.18-0.55)	
	5-9 years			79 (72%)	30 (28%)	0.35 (0.20-0.60)	
	10-14 years			67 (48%)	73 (52%)	1.0	
Ethnic origin	foreign-born	***subtotal foreign-born***		***71 (56%)***	***76 (44%)***	***4.6 (2.4-8.8)***	
		in NL since	0-6 months	31 (65%)	17 (35%)	2.3 (1.0-5.2)	1.4 (0.54-3.7)
			7-30 months	18 (40%)	27 (60%)	6.3 (2.8-14.3)	3.9 (1.4-10.7)
			> 2.5. years	22 (41%)	32 (59%)	6.1 (2.8-13.4)	2.7 (1.0-6.8)
	Dutch born	both parents Dutch born		63 (81%)	15 (19%)	1.0	
		2d generation immigrant		124 (70%)	54 (30%)	1.8 (0.96-3.5)	1.4 (0.63-2.9)
BCG	BCG-vaccinated			67 (54%)	57 (46%)	1.8 (1.2-2.9)	
	No BCG vaccination or unknown			191 (68%)	88 (32%)	1.0	
Type of TB	PTB + PETB	***subtotal PTB + PETB***		***162 (66%)***	***88 (34%)***		
		ZN positive		6 (18%)	27 (82%)	17.8 (6.6-48.2)	7.4 (2.4-23.0)
		ZN negative, culture positive		21 (38%)	34 (62%)	6.2 (3.0-12.7)	3.0 (1.2-7.2)
		culture negative or unknown		135 (86%)	22 (14%)	0.67 (0.36-1.3)	0.60 (0.31-1.2)
	ETB	***subtotal ETB***		***96 (61%)***	***62 (39%)***		
		culture positive		5 (11%)	83 (89%)	30.9 (10.9-87.1)	11.6 (3.6-37.4)
		culture negative or unknown		91 (80%)	23 (20%)	1.0	1.0
Culture	not done or unknown			184 (87%)	28 (13%)	10.4 (6.3-17.0)	2.6 (1.2-5.3)
	culture performed			74 (39%)	117 (61%)	1.0	1.0

*Definition of abbreviations*: *CI* confidence interval, *OR* odds ratio, *BCG* Bacille Calmette-Guérin, *PTB* pulmonary tuberculosis, *ETB* extrapulmonary tuberculosis, *PETB* combined pulmonary and extrapulmonary tuberculosis, *NL* Netherlands, *ZN* Ziehl Neelsen microscopy of sputum. *Categories in italics are subtotals and not used in the regression analysis.*

^*#*^*Covariates used in the multivariate regression analysis: Age group, ethnic origin, BCG, type of TB and culture.*

## Discussion

Our study shows that in the Netherlands the number of children with TB and children with LTBI declined with more than 50% over the last decade. The trend in childhood TB incidence mirrors the overall decline in TB incidence. Nearly two thirds of children with TB were detected through active case finding in contrast with 16% of adults. However, among foreign-born and second generation immigrant children no significant decline in TB incidence was observed and foreign born children were more likely to be detected through the development of symptomatic TB disease.

The quality of the diagnosis of TB in children seems to be high compared to other low prevalence countries [16-21], with culture confirmation rates of TB in children detected passively up to 68%, only slightly lower than in adults (74%). Treatment results exceed the international standards of 85% successful treatment completion, both for curative and preventive treatment. For every child diagnosed with TB, on average two children with LTBI were identified and started on preventive treatment. Assuming a life time risk for progression to TB disease of 5-10% [22] and an effectiveness of preventive treatment of 70% [23] 35–65 child TB cases are likely to have been prevented in the period 2005–2012. However, for 37% of child TB cases there was at least one ‘missed opportunity for prevention’ identified, among which absence of BCG vaccination in children from target groups was the most frequent.

Our study gives a representative picture of both notified child TB and LTBI cases in the Netherlands. The data represent both symptomatic cases detected in the curative sector as cases detected and managed ambulatory in the public health sector by the MPHSs. Sub analysis shows that foreign-born children with TB were less likely to be found through active case finding and that these children more often had advanced culture-positive TB disease.

A limitation of our study is that the data are retrieved from the routine TB surveillance registry and are not collected systematically with a strict research design. The Dutch surveillance system is generally regarded as sound and representative of TB incidence [24], but specific data e.g. on BCG vaccination status and laboratory results may be less complete. We chose presence of BCG scar or proof of vaccination as a proxy for BCG status, but in passively detected TB cases BCG status is not relevant for the clinical management and therefore more likely to be missing. Thus BCG vaccination coverage among the child TB cases may be underestimated.

We observed an overall culture-confirmation rate of 32% in TB diagnosis in children. Similar low rates have also been reported recently in other low-endemic countries [2,17,18,25,26]. However in our study, the culture confirmation rate among children with advanced, passively detected TB was high and comparable to the rate in adults. This is comparable or higher than rates reported in other studies ranging from 40%-64% among cases from whom material for culture was obtained [2,4,19]. Moreover, nearly two thirds of child TB cases were detected through active case finding, mainly through contact investigation. In this group the culture-confirmation rate was low because culture was not performed in 65% of the cases, but confirmation was also low in cases where culture was attempted. This reflects the difficulty to obtain culture confirmation in early TB disease in children as well as the Dutch consensus that the combination of obvious exposure, immunologic evidence of TB infection and radiological signs on the chest X-ray suggestive for primary TB infection allows for accurate diagnosis in this low-incidence setting. The drug susceptibility pattern of the putative source case guides the choice of treatment. Not endeavoring to obtain bacteriological confirmation through invasive methods in these circumstances is considered an effective, child-friendly and cost-saving approach.

TB incidence rates did decline among Dutch children but did not decline proportionally among child immigrant populations. In 2012, second generation migrant children had a 12 times higher risk to be diagnosed with TB than native Dutch children and foreign-born children had an 80 times higher risk. This reflects the high risk of transmission in the home countries and continuous risk of transmission among immigrant population groups after arrival in the Netherlands. Our study shows several opportunities for prevention of TB among these children. Varying results have been found in other high income countries with a low prevalence of TB [19,25,27]. Universal coverage of BCG vaccination in target groups would have most impact on the occurrence of TB, as nearly 40% of children with TB belonging to the target groups of BCG vaccination were not vaccinated, of whom 61% younger than 5 years of age. Assuming a 50% risk reduction following BCG-vaccination [12], at least 40 cases, 10% of the total case load in children, could have been prevented. Furthermore, recently evidence emerged that BCG may protect against TB infection, as well as the development of TB disease after infection [28,29]. BCG coverage is estimated to be below 60% in the Netherlands [30]. Based on a cost effectiveness analysis [31] the National Health Council recommends to continue targeted BCG vaccination among new-born children of parents originating from TB endemic areas with a WHO estimated incidence of > 50 per 100.000 population. To enhance the coverage the National Health Council recently recommended to include BCG vaccination in the national expanded childhood immunization program [30]. Our data support this recommendation.

Other interventions with potential impact on the occurrence of TB specifically among immigrant children, are improved contact investigation practices and screening and preventive treatment for LTBI on entry screening. Currently, only non-BCG vaccinated children younger than 12 years of age are tested for LTBI on entry in the Netherlands. However, more than half of the foreign-born children cases developed TB within 2.5 years in the Netherlands. It is likely that a considerable proportion of these children could have been detected with LTBI on entry in the Netherlands. Therefore the feasibility and cost-effectiveness of preventive treatment in immigrant children needs to be studied.

## Conclusion

TB case finding and TB treatment among children in the Netherlands is comparatively well managed, as evidenced by the fact that in the majority of cases a rapid diagnosis is made at an early stage through contact investigation and risk group screening. Furthermore, treatment success rates are high and no relapse cases were documented. It is likely that considerable TB morbidity and mortality among children in the Netherlands has been prevented through active case finding and preventive treatment of infected cases. However, excess mortality and morbidity may be further prevented through improved implementation of existing preventive measures such as targeted BCG vaccination and diagnosis and treatment of LTBI among foreign born children, and exploring feasibility and (cost) effectiveness of screening and preventive treatment of LTBI in children from TB high endemic countries.

## Competing interests

The author(s) declare that they have no competing interests.

## Authors’ contributions

CE designed the study, performed the statistical analysis, and drafted the manuscript. SK helped to draft the manuscript and gave important input on the interpretation of the results in the discussion. ES participated in the collection and cleaning of the data and contributed to drafting of the manuscript. GdV conceived of the study, participated in its design and helped to draft the manuscript. SvdH participated in the design, assisted with the statistical analysis and helped to draft the manuscript. All authors read and approved the final manuscript.

## Pre-publication history

The pre-publication history for this paper can be accessed here:

http://www.biomedcentral.com/1471-2334/14/295/prepub
